# Plant Sterols and Stanols for Pediatric Patients with Increased Cardiovascular Risk

**DOI:** 10.3390/children11010129

**Published:** 2024-01-20

**Authors:** Cristina Pederiva, Giacomo Biasucci, Giuseppe Banderali, Maria Elena Capra

**Affiliations:** 1 Clinical Service for Dyslipidemias, Study and Prevention of Atherosclerosis in Childhood, Pediatrics Unit, ASST-Santi Paolo e Carlo, 20142 Milan, Italy; 2 Centre for Pediatric Dyslipidemias, Pediatrics and Neonatology Unit, Guglielmo da Saliceto Hospital, University of Parma, 29121 Piacenza, Italy; m.capra@ausl.pc.it; 3 Department of Medicine and Surgery, University of Parma, 43126 Parma, Italy; 4 Department of Translational Medical and Surgical Sciences, University of Parma, 43126 Parma, Italy

**Keywords:** plant sterols, plant stanols, cardiovascular prevention, childhood, hypercholesterolemia

## Abstract

The atherosclerotic process begins in childhood and progresses throughout adult age. Hypercholesterolemia, especially familial hypercholesterolemia (FH) and metabolic dysfunctions linked to weight excess and obesity, are the main atherosclerosis risk factors in pediatric patients and can be detected and treated starting from childhood. Nutritional intervention and a healthy-heart lifestyle are cornerstones and first-line treatments, with which, if necessary, drug therapy should be associated. For several years, functional foods enriched with plant sterols and stanols have been studied in the treatment of hypercholesterolemia, mainly as nutritional complements that can reduce LDL cholesterol; however, there is a lack of randomized controlled trials defining their long-term efficacy and safety, especially in pediatric age. This review aims to evaluate what the main published studies on sterols and stanols in pediatric subjects with dyslipidemia have taught us, providing an updated picture of the possible use of these dietary supplements in children and adolescents with dyslipidemia and increased cardiovascular risk. Nowadays, we can state that plant sterols and stanols should be considered as a valuable therapy in pediatric patients with hypercholesterolemia, bearing in mind that nutritional and lifestyle counseling and, when necessary, pharmacologic therapy, are the cornerstones of the treatment in developmental age.

## 1. Introduction

**Figure 1:**
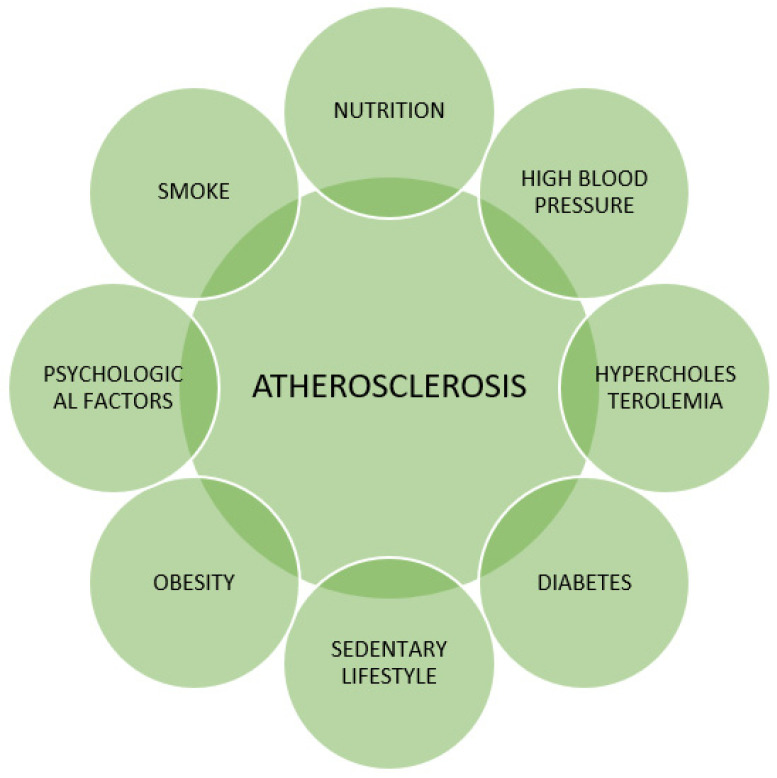
The main atherosclerosis risk factors are adapted from [5].

Coronary heart disease (CHD) is one of the main causes of mortality and morbidity worldwide, and atherosclerosis is its leading cause [1]. It is globally accepted that the atherosclerotic process starts even before birth [2] and progresses throughout childhood and adulthood, if not promptly detected and treated [3]. Atherosclerosis is a multifactorial process, and exposure to conditions linked to elevated cardiovascular risk accelerates and worsens the atherosclerotic cascade [4]. The main cardiovascular risk factors are summarized in Figure 1 [5].

Hypercholesterolemia, especially in its familial form, is a major atherosclerosis risk factor. Familial hypercholesterolemia (FH) is one of the most common inherited diseases, and its detection and treatment, when started in childhood, is a fundamental cornerstone in cardiovascular risk prevention [6]. Weight excess is another important cardiovascular risk factor. In the last decades, overweight and obesity have epidemically increased, posing a serious public health threat both for pediatric and adult subjects [7]. Nutritional and lifestyle interventions are milestones in the prevention and treatment of both these risk factors. However, functional foods use and/or drug therapy are often necessary in addition to this approach, to lower global cardiovascular risk [8]. Attention to plant sterols and stanols has increasingly grown due to their lipid-lowering effect. Plant sterol’s role in the treatment of pediatric patients with hypercholesterolemia had been considered more than forty years ago, but data on its efficacy and safety have been considered insufficient [9]. This review aims to evaluate what the main published studies on sterols and stanols in pediatric subjects with dyslipidemia have taught us, providing an updated picture of the possible use of these dietary supplements in children and adolescents with dyslipidemia and increased cardiovascular risk. The MEDLINE–PubMed database was searched to collect and select publications from 1990 to 2023. The search included randomized placebo-controlled trials, controlled clinical trials, double-blind, randomized controlled studies, and systematic reviews. The following combinations of keywords were used: “phytosterols” OR “stanols” AND “pediatric” OR “childhood” OR “adolescence” AND “cardiovascular risk” OR “hypercholesterolemia” OR “dyslipidemia” OR “familial hypercholesterolemia”. We also performed a manual search of the reference lists of the selected studies. The search was performed independently by two researchers. The search was limited to English-language journals and full papers only.

## 2. Plant Sterols and Stanols

Plant sterols can be defined as steroid alkaloids that have a different side chain with respect to cholesterol, whereas plant stanols are 5-alfa-saturated derivatives of plant sterols [9]. They cannot be synthesized by humans, so they must be taken through foods or nutraceutical compounds [9]. Plant sterols and stanols act as bioactive compounds with activity similar to that of cholesterol in mammals. Their cholesterol-lowering effect is due to their ability to interfere with cholesterol absorption and transport in the gut, mainly with a competitive mechanism [10].

**Figure 2:**
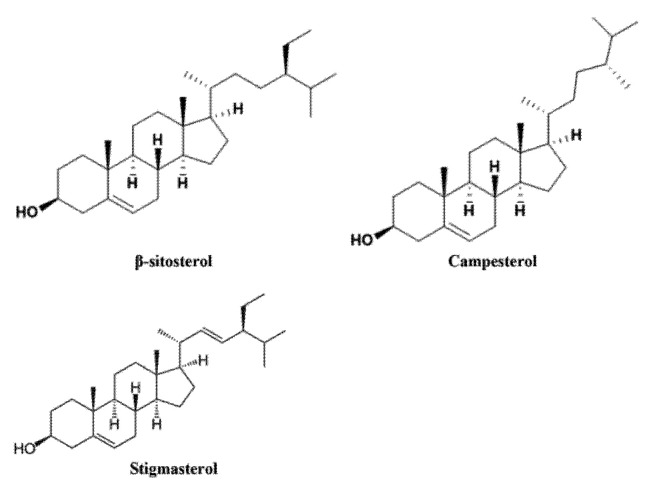
Chemical structure of main phytosterols, adapted from [14].

Plant sterols bioavailability in humans is generally low and it depends on various factors, including plant sterols type (sterols or stanols, esterified or not), plant sterols chemical structure (campesterol has higher absorption rates than beta-sitosterol) [11], processing (refining and hydrogenation of vegetable oils reduce sterols amount) [12], food preparation (boiling reduces plant sterols amount) [12], plant sterols source [13] and the co-existence of other nutrients in the food item (fibers, emulsifiers containing soy lecithin) [14]. The fact that plant sterols’ bioavailability is not constant is an important bias in studies testing their effects in humans. The main phytosterol chemical structure is shown in Figure 2.

They can be commonly found in food: cereals are the main stanols natural source, whereas plant sterols can be found also in vegetables, fruits, oils, and seeds, as shown in Table 1 [15,16] and Table 2.

Specific dietary patterns can provide different daily amounts of phytosterols [18]. Jaceldo et al. examined dietary intake of plant sterols in 816 adult subjects who took part in the Adventist Health Study-2. Total phytosterols daily intake was 263 mg/day for vegetarians and 428 mg/day for vegans. However, in this cohort plasma plant sterols concentrations and cholesterol plasma levels did not differ according to the dietary pattern [19]. The dietary approach to stop hypertension diet (DASH) can provide up to 550 mg of phytosterols/day [20], and the same daily intake can be reached through a Mediterranean diet [21]. Ras et al. conducted an observational study on a cohort of 35597 Dutch adult subjects who were participating in the European Prospective Investigation into Cancer and Nutrition-the Netherlands Study (EPIC-Nl) to assess dietary phytosterols intake. They found that the average energy-adjusted phytosterols daily intake was 296 mg [22]. Some authors report that the average daily intake of plant sterols and stanols is 500 mg [14]. Reaching a plant sterol and stanols concentration sufficient to have a significant effect on lipid profile only by diet is extremely difficult, given their poor gut absorption. Therefore, they should be added to food products to reach a higher concentration. When phytosterols are taken through enriched foods, in vivo safety and bioactivity can be influenced by various factors [23]. In a review, Cusack et al. discussed the effect of the food matrix on phytosterols effects [24]. The best food matrix through which phytosterols can be delivered are those rich in fat, to enhance their solubility. When low-fat matrices are used, emulsifiers (such as lecithin) addition can increase phytosterols solubilization [24]. Margarine is one of the most widely used food matrices to vehicle phytosterols as it has a high-fat content, thus enhancing phytosterols solubility; due to its flexibility in different food preparations, margarine intake usually obtains good dietary compliance and adherence. Mayonnaise food matrices have been studied as well, and those made with diacylglycerol seem to be more effective than those derived from triglycerides. Also, yogurt can be considered a relevant food matrix; it may also be effective and versatile, as it can be consumed as a snack or during a meal. Tortilla chips, ground meat, and chocolates have been studied as phytosterols delivering food matrices, but further studies are needed to better understand how successfully phytosterols can be incorporated in these food matrices [24]. The European Food Safety Authority (EFSA) has claimed that products containing at least 0.8 g of plant sterols per portion can have positive health effects in terms of lipid-lowering activity [25]. More recently, EFSA has stated that a 3 g daily intake of plant sterols for at least three consecutive weeks can help reduce plasma cholesterol levels by up to 11.3% [26]. Plant sterols or stanols addition in food must be indicated by the producers, according to European Community reg. CE 1169/2011 [27]. Concerning plant sterols safety and tolerability profile, possible interference with liposoluble vitamins has been hypothesized, with a subsequent reduction in these vitamins’ absorption and a reduction of carotenoid plasma levels; however, there is no evidence yet of low carotenoid and vitamin levels due to concomitant plant sterols consumption [28]. The main real counterindication to plant sterols use is sitosterolemia, a rare genetic disease with recessive autosomal inheritance caused by a misfunctioning of ABCG5 and ABCG8 and characterized by an increased plant sterols absorption and premature atherosclerosis [28]. An increase in sitosterol and LDL-C plasma levels, with consequent increased CHD risk, has been described also in subjects heterozygous for a “loss of function” variant of ABCG5/8: supplementation with plant sterols can be dangerous in this category of subjects as well [28].

## 3. Plant Sterols and Stanols in Human Disease and in Cardiovascular Prevention in Adult Subjects

Evidence supports the idea that plant sterols can have multiple beneficial roles in human health [29]. Plant sterols and stanols’ role in the treatment of different human diseases has been widely studied in many scientific papers. We focus on studies aimed at evaluating plant sterols’ possible anti-inflammatory effect on cells or tissues, linking their consumption to a reduction in tumor development risk, and analyzing their effect on gestational diabetes mellitus and its consequences on newborns’ health [11,30,31,32]. Plant sterol and stanols’ most widely studied role is surely their cholesterol-lowering effect and their role in cardiovascular risk prevention; they can reduce plasma total- and LDL-C levels, thus exerting healthy heart effects; indeed, this is one of their most studied and relevant possible implications in the clinical practice [33,34]. Moreover, plant sterols may have a positive epigenetic effect through the modification of gene expression that encodes proteins carrying sterols, thus obtaining an increase in cholesterol efflux from enterocytes to the gut lumen [35].

The interest in plant sterols’ healthy-heart effects dates back five decades ago: in 1977, Lees et al. conducted a survey on 46 adult subjects with hyperlipoproteinemia type II. These subjects were given a preparation containing plant sterols and this resulted in a 12% reduction of plasma total cholesterol concerning basal values [36]. In the following years, many studies have investigated plant sterols’ effect on lipid profile in adult subjects, confirming these preliminary data [37,38,39]. Miettinen et al. published a study in the New England Journal of Medicine on subjects treated with a daily dose of 2.6 g plant sterols enriched margarine: they reported a 14.1% reduction in LDL-C plasma levels in the treated subjects when compared to the placebo group, with no modification of HDL-C values [37]. Parraga Martinez et al. conducted a study on 182 adult subjects with hypercholesterolemia, who were given plant sterols 2 g each day for 12 months. Plant sterol’s lipid-lowering effect was confirmed in this study, which had a longer intervention period compared to previous ones [40]. In a recent systematic review and meta-analysis, an average LDL-C reduction of 0.55 mmol/L was reported, highlighting the importance of plant sterols dosage and the food matrix as main determinants of LDL-C reduction [41]. In recently published studies including cohorts of patients with FH, a low-fat diet combined with plant stanols resulted more effective in plasma total and LDL cholesterol values reduction if compared to dietary intervention alone (−0.62 mmol/L and −0.58 mmol/L respectively) [42]. Up to now, there are no randomized, controlled clinical trials that state plant sterols and stanols have beneficial effects in terms of cardiovascular disease treatment and prevention. In particular, there is no evidence of the significant effect of plant sterols and stanols intake on surrogate atherosclerosis markers, such as carotid intima-media thickness (IMT) and flow-mediated dilation (FMD) [43,44]. In a controlled study in healthy adult subjects, daily consumption through natural sources of up to 449 mg of plant sterols (in a diet implying 2000 kcal daily intake) was not related to a modification in plasma LDL-C concentration [45]. However, most of the evidence supports phytosterols and plant stanols’ positive effects in terms of cardiovascular risk reduction [46].

Recent EAS/ESC guidelines advise the consumption of functional foods enriched with plant sterols and stanols for subjects at increased CHD risk, including children with FH [47]. EAS also stated plant sterols and stanols safety and absence of toxicity at the prescribed dosages. In the Consensus document [47] the use of these functional foods was advised for specific categories of patients, as summarized in Table 3.

## 4. Plant Sterols and Cardiovascular Prevention in Pediatric Subjects

### 4.1. Familial Hypercholesterolemia

FH is a very common inherited disease, affecting approximately 1 out of 200–250 subjects in the general population [6]. Detection and treatment of subjects with FH starting from childhood helps “gaining decades of life”, as stated in the EAS Consensus Document [6]. Nutritional and lifestyle interventions are the cornerstone of FH treatment in pediatric patients, but in most cases, pharmacologic therapy should be started if dietary treatment is not effective or in case of severe hypercholesterolemia, so as to lower cholesterol burden [48]. In this context, the use of functional foods can be considered for a short period in those patients who are not old enough to start drug therapy, or who do not tolerate it, or in a combined therapy [49]. Plant sterols and stanols consumed in pediatric patients with FH are safe and it is a treatment applicable to all family members with FH, as a supportive therapy [50]. The study conducted by Becker et al. is one of the first ones evaluating the effects of plant sterols on children with FH. It is an interventional study involving 7 children with FH who were supplemented with sitosterol (2 g, 3 times a day for 3 months) compared to dietary intervention: nutritional treatment resulted in a 4.5% reduction of TC and 6.6% LDL-C with respect to basal values, whereas the addition of sitosterol led to a better lipid lowering effect (17% LDL-C reduction) [51]. A few years later, Gylling conducted a double-blind cross-over trial in pediatric patients with FH who were given spread with 3 g sitosterol esters per day. In this study, lipid profile was evaluated at baseline (on a lipid-lowering diet), and after 6 weeks of supplementation plant sterols supplementation allowed a 15% LDL-C reduction with respect to basal values, and the proposed spread was well accepted and tolerated by all patients [52]. In 2002, Amundsen reported a 10.2% LDL-C reduction after a 1.6 g/day plant sterols supplementation in FH children on the Step One Diet. In this 8-week double-blind randomized trial, he reported also a 7.4% Apolipoprotein B reduction. Ketomaki et al. analyzed lipid profile and red blood cell lipid content modification in a population of children with FH who were given spread at low or high plant sterols content for five weeks. LDL-C was reduced by 12%, whereas plasma HDL-C and red blood cells’ total cholesterol content were not significantly modified [53]. De Jongh et al. evaluated the effect of consumption of spread containing 2.3 g plant sterols per day in pre-pubertal children. In this four-week double-blind, cross-over placebo-controlled trial, both lipid profile and flow-mediated dilation (FMD) were evaluated, but only LDL-C resulted significant reduction in the intervention group with respect to placebo (14%). Children with FH had impaired FMD at baseline. After intervention, their FMD was not significantly modified, despite LDL-C reduction. This could be due to the short duration of treatment [54]. In 2006, Wiegman et al. reported a 9.2% LDL-C reduction in the intervention group (FH children who had a daily intake of 2 g plant sterols-enriched yogurt) with respect to the control group. No improvement in endothelial function and no modifications of plasma HDL-C and triglycerides levels were reported after a 4-week intervention [55]. Guardamagna et al. [56] evaluated the lipid profile after consumption of yogurt enriched with plant sterols in children with various forms of dyslipidemia. In this open-label 12-week trial, LDL-C reduction in children with FH was 10.7%, and yogurt enriched with plant sterols was well tolerated. Garoufi et al. conducted a larger population study, involving 64 pediatric patients with FH on a low-lipid diet. They were given yogurt enriched with 2 g plant sterols daily. LDL-C reduction was 13% concerning basal values, whereas no variations in HDL-C, triglycerides, and lipoprotein (a) plasma values were reported [56].

The use of functional foods enriched with plant sterols for pediatric subjects with FH has been included both in the 2011 National Cholesterol Education Program (NCEP) guidelines for LDL-cholesterol reduction [4] and in the EAS Consensus [10,47]. In the past decades, the use of functional foods enriched with plant sterols or stanols in pediatric patients with FH has been analyzed in various studies, even if most of them are either not so recent or involve a small size study sample [57,58]. Natural plant sterols daily intake has been correlated with an LDL-C lowering effect [56]. Most studies highlighted that the consumption of functional foods enriched with plant sterols and stanols can lead to a 10% average reduction of plasma total cholesterol and LDL-C, with respect to basal values. In particular, a 1.6 to 2.3 g plant sterols daily supplementation was effective in improving lipid profile in children with FH on CHILD I or CHILD II nutritional treatment [59,60,61]. The lipid-lowering effect is dose-dependent for supplementation up to 3 g per day, whereas above this threshold there is a so-called “plateau effect” [62]. The main matrices used for plant sterols supplementation are margarine, spreads, and yogurt, and, with lower frequency, semi-skimmed milk, and bread; this is in line with what is advised in the main trials/documents that have analyzed kinetics and absorption of plant sterols both on preclinical and in clinical practice [34,35]. What is more, it is worth remembering that plant sterols and stanols must be consumed during the meal, to grant a better uptake [35]. Yogurt and semi-skimmed milk are the best matrices, and they are easily consumed at breakfast or as breaks also by children and adolescents.

Trials analyzing co-administrations of statins and plant sterols are mainly conducted in adult subjects [59,62]. Vuorio et al. analyzed a cohort of pediatric patients with hypercholesterolemia on simvastatin therapy who were given margarine enriched with 2.24 g/day of plant sterols for six weeks: LDL-C reduction was 20% of basal values in both groups, independently from statin dosage [50]. There are no trials on plant sterols/statin combined therapy in children and adolescents and this could be an important topic for future studies.

Results on plant sterols and stanols lipid-lowering effect have been reported, as summarized in Table 4.

The main biases of the reported studies are the small number of patients enrolled and the average short duration of plant sterols supplementation. The great variability in the dosages used is a confounding factor as well. Further studies are needed to test the effect of plant sterols and stanols supplementation in pediatric patients with FH in larger study samples and for longer periods. The persistence of the lipid-lowering effect after the supplementation discontinuation should also be tested.

### 4.2. Other Dyslipidemias

The effect of plant sterols and stanols supplementation on lipid profile has been tested also in pediatric patients with hyperlipidemia other than FH. Williams et al. evaluated the effect of a 1-week supplementation with plant sterols esters and bran fiber on the lipid profile of preschool-age children. Participants in this study were randomized to receive either plant-sterol-ester spread (containing 3 g of plant sterols per day for four weeks) or dietary fiber (5 g per day for two weeks and then 10 g per day for a further two weeks). Supplementation with plant sterols resulted in a 12.4% and 15.5% reduction of plasma total cholesterol and LDL-C levels from baseline, respectively. No short-term side effects were reported and plant sterols consumption was generally well accepted [65]. Tammy et al. in the STRIP project evaluated plant sterols’ effect on lipid profile and their safety in healthy 6-year-old children already on a low-lipid diet, who were given a spread containing 1.5 g plant sterols per day. LDL-C plasma level was reduced by 7.5%, whereas no modifications in HDL-C, triglycerides, and alfa-tocopherol/LDL-C plasma values were reported. The proposed spread was generally well accepted and tolerated by young patients [66]. Matsuyama et al. evaluated the effect of plant sterols (0.4 g per day) on additional bread intake over 16 weeks in children with dyslipidemia. An 11.2% LDL-C reduction was obtained in this group of children, compared to 6.3% in the general population [67]. In an interventional study conducted by Guardamagna et al. in 2011 [56], 58 pediatric patients with hypercholesterolemia, aged 8–16 years, were given yogurt supplemented with 1.6 to 2 g plant sterols daily, or regular yogurt for 12 weeks; 32 patients had FH, 13 familial combined hyperlipidemia (FCH), and 13 undefined hypercholesterolemia (UH). Plant sterols supplementation resulted effective in reducing total- and LDL-C plasma levels in all patients, but the effect was higher in the group with UH (13% and 10.2% total cholesterol reduction, and 16% and 12.4% LDL-C reduction in the UH group and FH group, respectively). In the FCH group, there was a tendency towards lower lipid values during the treatment with plant sterols, without statistical significance. The possible explanation for the higher effect observed in UH compared to FH patients may be the different genetic backgrounds and pathophysiological mechanisms of FH and UH [56]. This result is consistent with the one by Ketomaki et al. on plant sterols supplementation in children with hypercholesterolemia with and without FH diagnosis [64]. In a randomized, double-blind cross-over trial, Ribas et al. recently reported a 10.2% LDL-C and 9.7% triglycerides plasma values reduction in children with dyslipidemia treated with milk enriched with 1.2 g plant sterols daily, with respect to the control group. The treatment was given for an 8-week period and no adverse effects were reported [68]. Tavares et al., in a study conducted in 2021, reported no reduction in LDL-C or triglycerides plasma levels after an 8-week supplementation with 2 g plant sterols daily in children with dyslipidemia [69]. The main studies on plant sterols and stanols’ effect on lipid profile in pediatric patients with dyslipidemia are summarized in Table 5.

As shown in Table 5, nutritional intervention with functional foods enriched with plant sterols or stanols exerts an LDL cholesterol-lowering effect also in patients with non-genetically determined dyslipidemia, with a mean reduction of 10–12% of basal LDL cholesterol plasma values. In some studies, cholesterol-lowering effect of plant sterols and stanols in subjects with FH has been compared to that seen in non-FH subjects [53,56], showing that the cholesterol-lowering effect was higher in the latter. This finding is consistent with the overall better response to nutritional intervention in subjects with non-genetically determined dyslipidemia. Tavares et al. do not report a significative reduction in LDL cholesterol plasma levels [69]: a possible explanation for this finding may be the fact that plant sterols were consumed as capsules and not in a food matrix or, as suggested by the authors themselves, to poor adherence to the proposed diet (NCEP Step Two Diet), which is very restrictive.

### 4.3. Weight Excess and Non-Alcoholic Fatty Liver Diseases

Non-alcoholic fatty liver disease (NAFLD) is a very common cause of chronic disease and it is a very severe complication of obesity and overweight in childhood. In adult subjects, the definition of NAFLD has been recently substituted with that of Metabolic-associated fatty liver disease (MAFLD), which better defines the tight connection between liver dysfunction and metabolic disorders [71]. This conceptual shift has been recently proposed for pediatric subjects as well [72]. Plant sterols and stanols can have beneficial effects in the treatment of patients with NAFLD. They can reduce inflammation, reduce liver steatosis, and modulate oxidative pathways. In particular, stigmasterol can lower liver triglycerides concentration and β-sitosterol can reduce liver cholesterol and increase liver polyunsaturated fatty acids (PUFA) content. All these actions, when combined, may exert an attenuative effect on NAFLD [73]. Animal models on the use of plant sterols for NAFLD treatment provided promising results [73], and this encouraged research in humans. In adult subjects, two recent clinical trials highlighted that daily plant sterols supplementation resulted in improvement in patients’ biological profile [74] and reduction of plasma LDL-C levels and systemic inflammation [75]. Further studies are needed to better analyze plant sterols’ effect on subjects with NAFLD, extended also to pediatric subjects. The plant sterols effect has been tested in children with obesity by Olivares et al. in a double-blind randomized controlled clinical trial involving 67 children with obesity. In the intervention group, daily intake of plant sterols-enriched milk (2.24 g of plant sterols daily) resulted in a significant reduction in plasma triglyceride levels for the control group [76]. Studies on MAFLD in pediatric patients are still not numerous. As we have previously stated, plant sterols and stanols seem to exert a modulatory effect not only on lipid metabolism but also on glucidic metabolism and the inflammation process, therefore, they could have a beneficial effect on subjects with MAFLD in childhood and adolescence.

## 5. Conclusive Considerations

Plant sterols and stanols have a proven lipid-lowering effect and their consumption is safe in humans [10]. Reaching a therapeutic concentration of plant sterols and stanols only through food is very difficult, given their poor intestinal absorption and their relatively low concentration in food items [14]. Therefore, the lipid-lowering effect of plant sterols and stanols can be better reached through the intake of food enriched with 1.5 to 3 g of plant sterols or stanols per day [77]. International guidelines for hypercholesterolemia suggest the use of plant sterols and stanols for adult and pediatric patients aged more than six years [4,10,47], though evidence of plant sterols and stanols lipid-lowering effect in pediatric patients is not as robust as in adults. Studies on plant sterols and stanols in pediatric patients have been conducted in small samples and for short periods, being rather scarce in the last decade. A possible explanation for this apparent lack of interest in plant sterols and stanols in pediatric subjects could be linked to the increasing use of drug treatments in pediatric patients with severe hypercholesterolemia, starting early in childhood. Studies on statin therapy in children and adolescents with hypercholesterolemia have proved to be safe and effective also in long-term follow-up [78,79] and, at present, statins are used and prescribed routinely in pediatric patients with FH. Studies on the combined effect of statins and phytosterol are few, with short-term design and on limited study population [59]. In a study on 21 adult subjects with hypercholesterolemia, the combined therapy of phytosterols and ezetimibe significantly enhanced the effects of ezetimibe on whole-body cholesterol metabolism and plasma LDL-C [80]. The use of phytosterols, alone or associated with statins or ezetimibe, in pediatric patients with mild hypercholesterolemia may represent an interesting field for further studies.

At present, plant sterols and stanols should be considered as a valuable therapy in pediatric patients with hypercholesterolemia, bearing in mind that nutritional and lifestyle counseling and, when necessary, pharmacologic therapy, are the cornerstones of the treatment in developmental age. Nutraceuticals should be prescribed under strict medical control and with a clear and effective alliance between pediatric lipidologists, patients, and their families, to avoid their improper use.

## Figures and Tables

**Table 1:** Average content of plant sterols and stanols in widely consumed foods; data expressed in mg/100 g of dry weight food (adapted from [15,16]).

Food	Plant Stanols	Plant Sterols
Sunflower oil	4	263–376
Olive oil	0.3–4	144–193
Corn	-	66–178
Wheat	17	45–83
Rice	3	72
Broccoli	2	39
Cauliflower	Traces	18–40
Almonds	-	143
Avocado	0.5	75
Passion fruit	-	44
Apple	0.8	12–18
Banana	0.8	12–18

**Table 2:** Percentage of plant sterols in oils, expressed as a percentage of total sterols fractions [17].

Type of Oil	β-Sitosterol	Campesterol
Corn-oil	55–67	7.2–8.4
Olive oil	75.6–90	2.3–3.6
Sunflower oil	56–63	7–13
Peanut oil	48–65	12–20

**Table 3:** Current recommendations on the use of plant sterols and stanols according to EAS [47].

Categories of Patients Eligible for the Use of Plant Sterols and Stanols
Subjects with elevated cholesterol levels at intermediate or low global cardiovascular risk who are not eligible for drug therapy. Subjects at high and very high cardiovascular risk who fail to achieve LDL-C targets on statins or are statin-intolerant (additive therapy). Subjects older than six years with familial hypercholesterolemia (FH).

**Table 4:** Main studies evaluating the effect of plant sterols and stanols supplementation on lipid profile in pediatric patients with FH.

Plant Sterols and Stanols in Pediatric Patients with FH			
Type of Study	Population and Intervention	Results	Year
Interventional study	7 prepubertal children (age 5.3 to 10.8 years) with severe heterozygous FH, were treated with sitosterol 2 g, three times a day for three months. Then combined therapy with sitosterol 1 g three times a day and bezafibrate 200 mg per day for three months.	Sitosterol lowered LDL-C (17%) Combination therapy of sitosterol and bezafibrate lowered LDL-C (50%) and TC (49%), with a 24-month lasting effect	1992 [51]
Interventional study	9 children with severe FH, aged 9–14 years. Intake of sitosterol pastilles (2 g 3 times a day) for 3 months, then 0.5 g 3 times a day of sitostanol for 7 months.	32% LDL-C reduction for sitostanol with respect to sitosterol	1993 [63]
Double-blind cross-over trial	1 child with HoFH and 14 with HeFH. Consume rapeseed oil margarine with or without 3 g/day of sitostanol esters.	15% LDL-C reduction in HeFH patients	1995 [52]
Intervention trial	24 children with HeFH, aged 3–13 years. Consume rapeseed margarine containing 2.24 g/day stanols concerning placebo for 12 weeks.	18% LDL-C reduction	2000 [59]
Double-blind, cross-over randomized clinical trial	38 children aged 7–12 years with definite or possible FH diagnosis. CHILD I diet and 1.6 g/day of stanols or placebo for 8 weeks.	7.4% plasma total cholesterol, and 10.2% LDL-C reduction	2002 [64]
Double-blind randomized crossover clinical trial	17 children with FH and 6 children with hypercholesterolemia, age 2–9 years, 2 g sterols/day enriched spreads for 5 weeks.	Stanol and sterol esters reduce the concentrations of plasma total cholesterol (9%) and LDL-C (12%)	2002 [53]
Double-blind cross-over trial	41 children with FH, Age 5–12 years. Plant sterols (2.3 g/day) enriched spreads versus placebo spreads	11% Total cholesterol reduction, and 14% LDL-C reduction	2003 [54]
Double-blind, randomized controlled cross-over trial	37 children with FH, aged 7–13 years. Intervention: spread enriched with sterol 1.2 g/day for 8 weeks.	9.1% Total cholesterol reduction, and 11.4% LDL-C reduction	2004 [60]
Double-blind cross-over trial	42 prepubertal children with FH Plant stanols enriched yogurt versus low-fat placebo yogurt for 4 weeks.	9.2% LDL-C reduction	2006 [55]
Interventional study	32 children with FH, 13 with FCHL, and 13 with undefined hypercholesterolemia Plant sterol-enriched yogurt for 12 weeks.	10.7% LDL-C reduction in the FH group	2011 [56]
Cross over trial	64 children with clinical familial hypercholesterolemia, age 4.5–15.9 years. CHILD II diet and yogurt with 2 g/day sterols daily for 6–12 months.	13% LDL-C reduction	2014 [61]

**Table 5:** Main studies evaluating the effect of plant sterols and stanols supplementation on the lipid profile of pediatric patients with dyslipidemia.

Plant Sterols and Stanols in Pediatric Patients with Dyslipidemia			
Type of Study	Population and Intervention	Results	Year
Open cross-over study	Healthy preschool-age children (2 to 5 years old). Interventions: spread with 3 g plant sterols per day for four weeks versus fiber supplementation for 4 weeks, one week of wash out, then cross-over	Plant sterols reduce plasma TC (12.4%) and LDL-C (15.5%)	1999 [65]
Randomized prospective double-blind cross-over trial	81 children, aged 6 years. 20 g of the child’s dietary daily fat intake was replaced with plant stanol ester mararine containing 1.5 plant sterols per day for three months.	Plant stanols consumption resulted in the lowering of total cholesterol (5.4%) and LDL-C (7.5%)	2000 [66]
Double-blind randomized crossover clinical trial	17 children with FH and 6 children with hypercholesterolemia, age 2–9 years, 2 g sterols/day enriched spread for 5 weeks.	Stanol and sterol esters reduce the concentrations of plasma total cholesterol (9%) and LDL-C (12%)	2002 [53]
Clinical trial	22 children with hyperlipidemia, age 6–17 years. Intervention: bread enriched with 0.4 g/day of plant sterols for 16 weeks	2.2% Total cholesterol and 6.3% LDL-C reduction	2007 [67]
Interventional study	32 children with FH, 13 with FCHL, and 13 with undefined hypercholesterolemia. Plant sterol-enriched yogurt for 12 weeks.	10.7% LDL-C reduction in the FH group	2011 [56]
Clinical trial	25 children with hyperlipidemia, age 11–17 years. Intervention: a combined emulsion of sterol plants, fish oil, and vitamin B providing plant sterols 1.3 g/day for 16 weeks	7.7% total cholesterol and 8.4% LDL-C reduction	2013 [70]
Double-blind cross-trial	28 dyslipidemic children aged 6–9 years: the intervention group received milk enriched with 1.2 g/day of plant sterols for 8 weeks.	5.9% plasma total cholesterol and 10.2% plasma LDL-C reduction	2017 [68]
Randomized, double-blind, cross-over trial	31 children, aged 6–12 years, with hyperlipidemia. Intervention: phytosterol 2 g/day capsule supplementation for 8 weeks associated with NCPED Step 2 diet.	No significant reduction in total cholesterol and LDL-C levels	2021 [69]

## Data Availability

Not applicable.

## Abbreviations

CE	Comunità Europea
CHD	Coronary Heart Disease
DASH	Dietary-Approach to Stop Hypertension
EAS	European Atherosclerosis Society
EFSA	European Food Safety Authority
FCH	Familial Combined Hyperlipidemia
FH	Familial Hypercholesterolemia
FMD	Flow Mediated Dilation
HOMA-IR	Homeostasis Model Assessment-estimated Insulin Resistance
IMT	Intima Media Thickness
LDL-C	Low Density Lipoprotein Cholesterol
LDL-R	Low Density Lipoprotein Receptor
MAFLD	Metabolic Associated Fatty Liver Disease
NAFLD	Non-Alcoholic Fatty Liver Disease
NCEP	National Cholesterol Expert Panel
NPC1L1	Niemann-Pick C1-Like 1
TC	Total Cholesterol
UH	Undefined Hypercholesterolamia
